# Rejection markers in kidney transplantation: do new technologies help children?

**DOI:** 10.1007/s00467-022-05872-z

**Published:** 2023-01-17

**Authors:** Licia Peruzzi, Silvia Deaglio

**Affiliations:** 1 Pediatric Nephrology Unit, Regina Margherita Department, City of Health and Science University Hospital, Piazza Polonia 94, 10126 Turin, Italy; 2 Immunogenetics and Transplant Biology Service, City of Health and Science University Hospital, Turin, Italy; 3 Department of Medical Sciences, University of Turin, Turin, Italy

**Keywords:** Kidney transplantation, Antibody-mediated rejection, Biomarkers, Cell-mediated rejection, Chronic allograft rejection, Cell free DNA, Extracellular vesicles, Liquid biopsy

## Abstract

Recent insights in allorecognition and graft rejection mechanisms revealed a more complex picture than originally considered, involving multiple pathways of both adaptive and innate immune response, supplied by efficient inflammatory synergies. Current pillars of transplant monitoring are serum creatinine, proteinuria, and drug blood levels, which are considered as traditional markers, due to consolidated experience, low cost, and widespread availability. The most diffuse immunological biomarkers are donor-specific antibodies, which are included in routine post-transplant monitoring in many centers, although with some reproducibility issues and interpretation difficulties. Confirmed abnormalities in these traditional biomarkers raise the suspicion for rejection and guide the indication for graft biopsy, which is still considered the gold standard for rejection monitoring. Rapidly evolving new “omic” technologies have led to the identification of several novel biomarkers, which may change the landscape of transplant monitoring should their potential be confirmed. Among them, urinary chemokines and measurement of cell-free DNA of donor origin are perhaps the most promising. However, at the moment, these approaches remain highly expensive and cost-prohibitive in most settings, with limited clinical applicability; approachable costs upon technology investments would speed their integration. In addition, transcriptomics, metabolomics, proteomics, and the study of blood and urinary extracellular vesicles have the potential for early identification of subclinical rejection with high sensitivity and specificity, good reproducibility, and for gaining predictive value in an affordable cost setting. In the near future, information derived from these new biomarkers is expected to integrate traditional tools in routine use, allowing identification of rejection prior to clinical manifestations and timely therapeutic intervention. This review will discuss traditional, novel, and invasive and non-invasive biomarkers, underlining their strengths, limitations, and present or future applications in children.

**Supplementary information:**

The online version contains supplementary material available at 10.1007/s00467-022-05872-z.

## Introduction

The current view of allorecognition and graft rejection mechanisms is that of a highly complex and articulated phenomenon, where inflammatory processes synergize with adaptive immune responses resulting in T or B cell activation. This complexity is incompletely captured by the traditional laboratory tools used for monitoring graft health, and therefore, rejection monitoring remains an unmet clinical need.

Current pillars of transplant monitoring include traditional markers, such as serum creatinine, proteinuria, and drug blood levels, implemented by immunological biomarkers such as donor specific antibodies (DSA) [1]. Perturbations of these biomarkers guide the indication for graft biopsy, which is still considered the gold standard for rejection diagnosis.

The limits of these markers and the invasiveness of repeated biopsies, particularly in children, could be overcome by integration with the information provided by rapidly evolving novel biomarkers, which are currently used in limited clinical settings, particularly in children [2].

In the pediatric setting, overall results of kidney transplantation remain unsatisfactory in the long run, in particular when passing through the risky years of adolescence. A recent study highlighted the excess of late acute rejection episodes in the NAPRTCS cohort [3], pointing out the need to increase the performances of surveillance modalities after the generally adopted schedules of intensive monitoring of the first 24 months.

## The ideal biomarker

The basic definition of a biomarker is that of a defined characteristic, objectively measured and evaluated as an indicator of normal or pathogenic biological processes or responses to an exposure or intervention, which can be derived from molecular, histologic, radiographic, or physiologic characteristics.

A number of biomarkers can be identified with diagnostic, monitoring, response, predictive, prognostic, safety, and susceptibility/risk values. The NIH-FDA Biomarker Working Group has recently defined the characteristics of an ideal biomarker in the BEST Resource. (Details and reference are in Supplementary Table 1.)

One of the main characteristics of a biomarker is the capability to identify patients with high disease probability, avoiding false over/under-rating of different independent cohorts. The diagnostic/predictive accuracy of a biomarker is therefore very important to allow its confident use and is generally expressed by specific metrics data: sensitivity and specificity and the related area under the receiver operator characteristic curve (see Supplementary Table 1).

The predictive value of the biomarker is the capacity to predict a clinically relevant event: the negative predictive value (NPV) allows excluding confidently the disease, while the positive predictive value (PPV) confirms the disease status. A monitoring and predictive biomarker should change significantly in response to treatment, while a prognostic biomarker should predict a clinical outcome regardless of treatment, meaning that it can be used to determine the risk of a bad prognosis.

Laboratory tests considering biomarkers should possess a number of requirements, including characteristics of robust reproducibility and interpretability, as well as external validation confirming good performance metrics.

One further step is the use of a biomarker as a surrogate end point, as a tool for early detection of a certain condition. In the field of transplantation, a certain value could be considered a surrogate end point of rejection and be the threshold to proceed with biopsy or directly to treatment [4].

## Traditional biomarkers in transplantation and their limitations

Creatinine is the most commonly employed measurement to determine kidney function. While widely available and cheap, it is neither specific nor sensitive and is often a late indicator of subclinical rejection. This is particularly true in children receiving large grafts, as demonstrated by the frequent finding of subclinical histological rejection evident in protocol biopsies, with no modification in serum creatinine [5].

Among other kidney function tests, proteinuria is routinely measured and is considered as being among the standard of care. Its performance as a biomarker for rejection is, however, low, in children: albuminuria and proteinuria are nonspecific signs of allograft injury with high sensitivity for graft loss but low specificity. In the large adult cohort described in [6], early detection of proteinuria > 1 g/24 h displayed low sensitivity and high specificity for graft loss at 5 years (AUC 0.64, sensitivity 0.10; specificity 0.95), with increasing accuracy over time (AUC 0.71, 0.73, and 0.77, respectively, at 1, 3, and 5 years) but still low sensitivity (from 0.16 at 1 year to 0.28 at 5 years) and good specificity (from 0.95 at 1 year to 0.96 at 5 years). Proteinuria had a negative predictive value (91–93%) at any time, but no specific analysis considered the full T cell–mediated rejection/antibody-mediated rejection (TCMR/ABMR) definition. No specific prospective studies have explored the performance of the protein/creatinine ratio as a biomarker for rejection in children.

### Surveillance protocol biopsy

At present, tissue biopsy remains the gold standard for assaying the health of the graft, although arguments on specificity and sensitivity of histology lesions and their predictive value, particularly when performed early after transplant, are reported in different studies. Protocol surveillance biopsies possess the advantage of an unbiased longitudinal approach, but represent invasive procedures, particularly in small children: are risky mainly for occurrence of adverse events, such as bleeding and artero-venous fistula, time-consuming, subject to pathology interpretation, and generally limited to the first 12–24 months after transplantation. The role of protocol biopsies as modifiers of long-term allograft survival in children remains debated, due to different induction and maintenance immunosuppressive regimens, timing of biopsy, and policy for management of subclinical rejection [2, 4, 7]. At the same time, the incidental finding of non-specific signs of chronic allograft damage without actionable inflammation is a problematic issue even in per cause biopsies. Subclinical TCMR signs are reported in a significant unexpected proportion of patients in several retrospective series [2], reinforcing the advantages of preemptive treatment on longer graft survival [8]. On the other side, in a recent prospective trial, children with stable serum creatinine at 6 months and Banff lesions from borderline to Ia, Ib, or IIa were not treated and GFR remained stable at 24 months, independently of biopsy findings, suggesting that surveillance biopsy at 6 months could be spaced out in stable low-risk patients [4].

A recent survey in adult centers in the USA [9] reported that surveillance biopsy policy is adopted in 46% of the centers, underlying the notion that noninvasive immunological monitoring is perceived as unsatisfactory. However, the notion of repeated histology monitoring remains controversial, arguing for the need for controlled studies.

In the Canadian PROBE multicenter study [10], adjunctive control post-treatment biopsies were generally performed after a diagnosis of rejection during surveillance biopsies, frequently highlighting persistent inflammation. These findings raise concerns that the common use of functional monitoring to adjudicate rejection resolution is likely insufficient and not sensitive enough to confidently consider the rejection episode properly treated and solved.

Altogether, these reasons argue for the need to find additional noninvasive or minimally invasive monitoring systems that can be performed easily, diffusely, and longitudinally over time to continuously patrol subclinical rejection, evaluate evolution of the immunological phenomena upon treatment, and to allow personalized optimization of therapy until a successful resolution is confidently reached.

### Drug level monitoring

Drug level monitoring is generally accepted as a biomarker of proper immunosuppressive drug use. It is routinely performed for tacrolimus, cyclosporin, everolimus, and sirolimus. Mycophenolic acid single sample trough concentration is not a good surrogate for overall exposure of the drug. These limits are overcome by mycophenolic acid area under the curve (AUC) estimation, which proved to be an effective tool, although less practical, particularly in children, due to the need for at least 2 or 3 concentration samplings for mycophenolate mofetil and 3 to 4 samplings for mycophenolic acid assay [11]. Steroid effective dose determinations are still lacking.

Fluctuations in tacrolimus blood levels in individual patients at a fixed dose over time are defined as intra-patient variability (IPV). High tacrolimus IPV in adult studies correlates with development of DSA, allograft dysfunction, rejection, transplant glomerulopathy, and late graft loss (reviewed in Kuypers) [12].

In the few pediatric studies, tac IPV correlates with de novo DSA development. However, in children, the correlation with rejection, function decline, and graft loss is weaker likely due to biases in defining cut-off values, cohort numerosity, and methodological differences [13, 14] (detailed in Supplementary Table 2).

Future perspectives should advocate expert systems to estimate drug exposure [15], novel techniques to evaluate multiple drugs simultaneously, and the transition to the concept of “time in therapeutic range”, already adopted in other fields, as more precise predictors of under-suppression and potential risk of allograft rejection [16].

### Donor-specific antibodies

Donor-specific antibodies (DSA) are antibodies developed by the transplant recipient against specific HLA antigens present on the donor kidney. Rejection mediated by DSA may be acute if the graft is exposed to rapid increases in high-titer DSA, which may be generated in sensitized recipients or which may represent de novo responses in non-sensitized patients who are non-adherent to immunosuppressive therapy. On the other hand, chronic rejection mediated by DSA is associated with a slower appearance of antibodies, which may be high or low titer and transient or persistent [17].

These antibodies may damage the kidney by causing multi-lamination of the peritubular capillary basement membrane or arteriopathy manifesting as intimal fibrosis [17]. It is well established that the development of de novo DSA (dnDSA) after kidney transplantation is linked to poor graft outcomes in both adults and children [18, 19]. The effects of the sequelae of chronic antibody-mediated rejection are more difficult to control.

For these reasons, dnDSA represent an established biomarker predictive of late acute antibody-mediated rejection, chronic antibody-mediated rejection, transplant glomerulopathy, and graft loss [1], demonstrated also in children [19, 20].

**Fig. 1:**
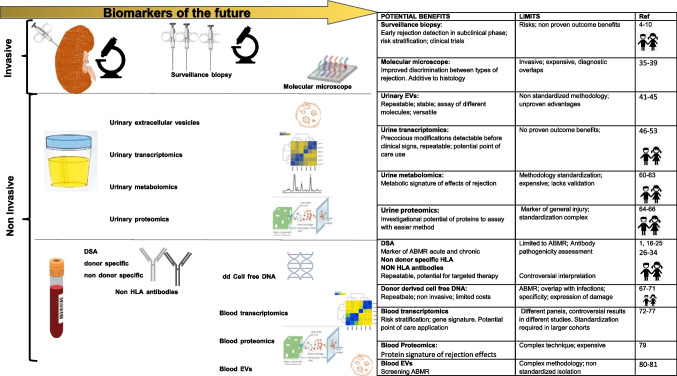
Traditional and novel biomarkers for kidney rejection diagnosis. Invasive and non-invasive biomarkers obtained from urine and from blood evaluated in children and adults at the present time, with benefits and limitations

Some firm points concerning the role of DSA in chronic allograft damage can be made:

1. DSA can be detected in the serum of kidney transplant recipients years prior to clinical graft dysfunction. For these reasons, it is important to monitor DSA routinely in the follow-up of transplant recipients, even though homogeneous protocols are lacking [19].
2. The clinical relevance of dnDSA relies on characteristics of the antibody itself, such as the IgG subclass, influencing the capability to bind complement and recruit effector cells through Fc receptor binding. DSA of IgG3 subclass bind C1q more efficiently, activate the classical pathway of complement cascade, and proceed more frequently to acute antibody-mediated rejection, while DSAs of IgG4 subclass are unable to bind complement and act mainly through the Fc receptor to amplify alloresponses [21].
3. In general, dnDSA formation has been associated with lower 10‐year graft survival, including in pediatric studies [19].
4. DSAs are generally measured by the single antigen Luminex technology, where the mean fluorescence intensity (MFI) is a proxy of the intensity of binding of the antibody contained in the patient serum to the beads. Consistently, higher MFI levels have been associated with impaired graft function, even though exceptions occur [22].
5. While there is intense investigation around therapeutic strategies aimed at reducing DSA levels [23], little is known about how to prevent initial DSA formation. Likewise, determination of risk factors for DSA development has not been fully characterized. Preliminary evidence suggests that the type of immunosuppressive therapy may impact DSA development [24]. Specifically, regimens based on calcineurin inhibitors appear less likely to be associated with DSA formation, compared to regimens based on mTOR inhibitors or lower mycophenolic acid levels [25] (Fig. 1).

### Non-donor-specific anti-HLA antibodies

The finding of non-donor-specific antibodies (NDSA), i.e., antibodies which are directed against HLA specificities other than the donor mismatched antigens, is a relatively common finding in post-transplant monitoring. While pre-transplant NDSAs have been demonstrated to have a detrimental role in graft outcome, the clinical significance of de novo NDSA (dnNDSA) is much more controversial [26]. Some studies have showed that post-transplant occurrence of dnNDSA in non-sensitized kidney graft recipients does not have an adverse effect on graft survival [19, 27], while others evidenced that patients developing dnNDSA have an increased risk of graft failure [28–30]. However, in all instances, patient cohorts have a short to medium post-transplant follow-up, with NDSA usually analyzed at a single time point [26], somewhat limiting the impact of these observations and arguing in favor of more structured studies (Fig. 1).

### Non-HLA antibodies

The observation that a significant subset of patients with histological features of antibody-mediated rejection (AMR) in the graft biopsy lacks evidence of dnDSA [31] was the starting point in the identification of non-HLA DSA [32].

Although the first reports connecting non-HLA antibodies and graft outcome were published in 2005 [33], their actual role in rejection remains debated. Among the different targets identified are antibodies against specific alloantigens such as MHC class I chain-related gene A (*MICA-Ab*) or B (*MICB*), or against autoantigens like endothelin-1 type A receptor (ETAR-Ab), perlecan, agrin, or vimentin, among others. Antibodies against angiotensin II type 1 receptor (AT1R-Ab) are among the most studied, including in pediatric patients [34] (Fig. 1).

## Biomarkers of the future (Table 1 and Fig. 1)

### Innovative diagnostics on tissue biopsies

“Tissue transcriptomics” relies upon analysis of mRNA transcripts on biopsy tissue fragments on a microarray technology. This technology provides a complementary quantitative and reproducible tool for deeper and more accurate graft biopsy analysis.

The microarray-based molecular diagnostic system MMDX®, known as “molecular microscope,” was set on an initial discovery study on 403 biopsies, and validated on a further independent cohort of 300 biopsies. The study identified a universal set of mRNA changes and specific sets of mRNA transcripts for TCMR and ABMR [35, 36]. Prominent transcripts of both rejections were interferon-γ (IFN-γ)-inducible genes in several types of cells. Based on these findings, different pathways were defined for TCMR-specific transcripts, including effector T cells, such as CTLA4, ICOS, and costimulatory molecules, macrophage infiltration, and activation molecules, such as IFN-γ-inducible CD80, CD86, and ANKRD22. ABMR selective transcripts, such as *CCL4*, *CXCL11*, and *CXCL9* resulted expressed only in NK cells or in endothelial cells. Some other markers proved specific for advanced phases of ABMR. Despite some overlap between the types of rejection, the association of transcripts with the subtypes of rejection is quite strong, as demonstrated by the multicenter INTERCOM study [37], which allowed the identification of ABMR in a high percentage of C4d and histology negative biopsies.

Another commercially available platform is the Nanostring® B-HOT panel, set up by the Banff Working Group in 2019, including 770 genes, pertinent to rejection, tolerance, viral infections, innate and adaptive immune responses, categorized on functional annotations for different pathways [38].

Based on the same technology, the GoCAR study defined a gene set capable of predicting kidney allografts at risk of progressive injury due to fibrosis [39]. In this study, the authors identified a set of 13 genes which proved independently predictive for the development of fibrosis at 1 year, superior to clinical and histology variables. The predictive value of this gene set was validated in an independent cohort from the GoCAR study and two independent, publicly available expression datasets (Fig. 1).

### Non-invasive novel urinary biomarkers

Urine matrix represents an appealing substrate for the development of a rejection biomarker, being the direct ultrafiltrate where, at least theoretically, an early perturbation of the usual asset can be assessed noninvasively. Some innovative methodological approaches adopted extracellular vesicle focused studies, assaying the lipid, protein, or mRNA content or combined transcript analysis with ELISA assay of target proteins.

Moreover, the development of new high-throughput techniques allowing the use of multiple”omics” assays rapidly led to different interesting studies evaluating mRNA transcripts, urinary metabolites, protein panels, and genomes in urine (Fig. 1 and Table 1).

**Table 1:** Detailed metrics of the studies included in the review

	Biomarker	Population	Test design	Sensitivity	Specificity	PPV	NPV	AUC
**Urinary extracellular vesicles**								
Sigdel 2015	Urine EVs proteomic	Adults	AR vs no rejection	–	–	–	–	–
Lim 2018	Urine EVs 5 proteomic biomarkers	Adults	TCMR vs stable	TSPAN1 and HPX 64%	72.7%	–	–	0.744
Jung 2021	Urine EVs 6 proteomic biomarkers:	Adults	Chronic ABMR vs stable renal function no rejection	APOA1 82.4% PIGR 70.6% TTR 76.5% HPX 58.8% AZGP1 64.7% CP 70.6%	83.3% 70.8% 76.5% 58.3% 66.7% 70.8%	77.8 63.2% 75% 50.0 57.9% 63.2%	87.0% 77.3% 81.8% 66.7% 72.7% 77.3%	0.926 0.762 0.846 0.728 0.743 0.855
Park 2017	Urine EVs T cell protein iKEA platform CD3	Adults	ACR vs no ACR	Training 92.8% Validation 63.3%	87.5% 100%	–	–	0.911 0.837
Fekih 2021	Urine exosome mRNA signature	Adults	Rejection vs no rejection TCMR vs ABMR	84.7% 87.5%	94.0% 82.9%	86.2% 77.8%	93.3% 90.6%	0.93 0.87
**Urine transcriptomics**								
Suthanthiran 2013	CD3ε mRNA + IP-10 mRNA + 18S rRNA	Adults	TCMR vs non-TCMR	79%	78%	–	–	0.850
Matignon 2014	26 mRNA + 18 s RNA (6 gene signature)	Adults	AR vs ATI ACR vs AMR	69% 65%	97% 81%	-	-	0.92 0.81
Sigdel 2019	BASP1, CD6, CXCL10, CXCL9, INPP5D, ISG20, LCK, NKG7, PSMB9,	Adults	AR vs bAR + HC	87.10%		–	–	0.9677
	RUNX3, TAP1 (uCRM Score)	Adults	AR vs BKVN + bAR + HC	76.92%		–	–	0.9111
Hricik 2013	Urine mRNA panel + urinary protein CXCL9 – CXCL10	Adults and **children**	AR vs no rejection	Granzyme mRNA 71% CXCL9mRNA 67% CXCL9 prot 85% CXCL10 prot 74%	82% 80% 81% 86%	65% 61% 67% 71%	85% 83% 81% 87%	0.73 078 0.85 0.77
Kaminski 2020	CXCL9 mRNA CRISPR	Adults	AR vs HC	93%	76%	–	–	0.91
Lorenzen 2011	miR-210	Adults	AR vs HC	74%	52%	–	–	0.70
Millán 2017	miR-155	Adults	AR vs non-AR	85%	86%	88%	100%	0.875
	CXCL10 mRNA	Adults	AR vs non-AR	84%	80%	90%	85%	0.865
Gielis 2021	miR-155-5p miR-615–39	Adults	AR vs non-AR	84.7% 88.7%	56.6% 24.1%	78.3% 67.7%	66.7% 54.2%	0.82 0.30
**Urine metabolomics**								
Blydt-Hansen 2014	Proline, PC:aa:C34:4, kynurenine, sarcosine, methionine sulfoxide,	**Children**	TCMR vs non-TCMR	83%	83%	97%	45%	0.880
	PC:ae:C38:6, threonine, glutamine, phenylalanine, alanine	**Children**	TCMR + borderline tubulitis vs	95% (training)	75% (training)	–	–	0.900 (training)
Banas 2018	Alanine, Citrate, dimethylamine (DMA), Glucose, Glucuronate, Hippurate, Lactate, Phenylacetylglutamine (PAQ), Trigonelline and Urea	Adults	Rejection vs non rejection	86% restrict model 86% Extended model	58 62	–	–	0.722 0.74
Yang 2020	Q-score: n 6 selected DNA, protein, and metabolite biomarkers consisting of cell-free DNA (cfDNA), methylated cell-free DNA (m-cfDNA), clusterin, total protein, creatinine, and CXCL10	Adults and **children**	Rejection vs non rejection	Finding 95 Valid 1 90.6% Valid 2 100%	Finding 99 Valid 1 91.6% Valid 2 6.3%	–	–	0.99 0.98 1
**Urinary proteomics**								
Kanzelmeyer 2019	79 peptides proteome	**Children**	cABMR vs no rejection	100%	75%	73%	100%	0.92
Mertens 2020	10 protein	Adults	ABMR vs no rejection	95% 95%	96% 76%	89% 33%	98% 98%	Training cohort 0.98 Validation cohort 0.88
**Peripheral blood transcriptomics**								
Christakoudi 2019	All genes 7 genes panel: *IFNG*, *IP-10*, *ITGA4*, *MARCH8*, *RORc*, *SEMA7A*, *WDR40A*	KALIBRE study Adults	TCMR vs stable	Training 85% Cross validation 67% Training 85% Cross validation 67%	Training 89% Cross validation 81% Training 93% Cross validation 85%	– –	– –	0.96 0.80 0.95 0.84
Zhang 2019	17 gene peripheral blood signature	GoCar study adults	Acute cellular rejection	Training set Testing set		79% 73%	98% 89%	0.83
Van Loon 2019	8-gene assay (*CXCL10*, *FCGR1A*, *FCGR1B*, *GBP1*, *GBP4*, *IL15*, *KLRC1*, *TIMP1*)	Biomargin	ABMR vs no rejection	Validation cohort 73%	75.7%	26.3%	96%	0.79
Roedder 2014	17 gene signature	AART study adults and **children**	AR vs no AR	AART143 82.3% AARt124 (ad + ch) 91.3% AART100 92.3%	90.6% 99% 93.4%	81.2% 95.4% 93.2%	91.5% 98% 93.4%	0.94 0.95 0.92
Van Loon 2020	*K-SORT*, *DUSP1*, *CFLAR*, *ITGAX*, *NAMPT*, *MAPK9*, *PSEN1*, *RYBP*, *NKTR*, *SLC25A37*, *CEACAM4*, *RARA*, *RXRA*, *EPOR*, *GZMK*, and *RHEB* Immucor	Adult Multicenter CENTAURE + Leuven	AR vs no-AR Any rejection vs no rejection	36% 31%	70% 69%	12% 34%	90% 65%	0.51 0.51
Friedewald 2018	Trugraf CTOT-08 study 57 gene expression profile	Adults	Sub AR vs stable with normal histology	64%	87%	61%	88%	0.85
**Blood proteomics**								
Cibrik 2013	17 proteins signature Lower expression (E-cadherin, EGF, erythropoietin receptor, growth regulated oncogene-alpha, interleukin 6, MCP-1 macrophage inflammatory protein 3-alpha, transforming growth factor β 1 and 2) Higher ((GM-CSF, interleukin 1 receptor 1, interleukin 12 p70, KIM-1, MIF, osteopontin, tumor necrosis factor receptor II, and vascular endothelial growth factor receptor	Adults	Stable vs rejecting	–	–	–	–	1
**Peripheral blood extracellular vesicles**								
Tower 2017	Plasma endothelial CD144 + EVs	Adults	ABMR vs no rejection	–	–	–	–	–
Zhang 2017	Plasma EVs (mRNA expression: combination score gp130, SH2D1B, TNFα, and CCL4	Adults	ABMR vs TCMR	77.8%	76.1%	76.5%	77.6%	0.796

Studies involving children are evidenced in bold

#### Urinary extracellular vesicles

Extracellular vesicles (EVs) are membrane vesicles released by all cell types with the capacity of transmitting signaling molecules to the surrounding environment and exerting a multitude of paracrine end endocrine effects in physiological and pathological conditions [40]. EVs are lipid bi-layered particles released from plasma membranes and are highly heterogenous for origin, size, and content. They carry proteins, lipids, nucleic acids, or other bioactive molecules specific to the parental cell of origin and are retrieved in plasma and urine in several forms ranging from 30–100 nm (exosomes) to 100–1000 nm (microvesicles) and with different density and protein markers. Accurate characterization in EV research studies is crucial, although still not standardized: the International Society for Extracellular Vesicles recommends size definition, quantification, characterization of surface and cytosolic proteins, and imaging for extracellular vesicle proper description and study comparison. These technical challenges render ample and multicenter clinical studies inevitably difficult.

In the field of transplantation, EVs circulating in plasma and secreted in urine are of high interest for the capacity to carry information originating directly from the grafted kidney or from the host immune system and are considered of enormous potential as non-invasive biomarkers.

Urinary EVs were studied in the setting of acute rejection on a proteomic profile: eleven proteins functionally involved in inflammatory and stress response were identified, with 3 exclusively urinary proteins more abundant in patients with acute rejection [41]. Other proteomic studies identified urinary protein biomarkers associated with acute cellular rejection (ACR), able to discriminate between acute cellular or antibody-mediated rejection and chronic active antibody rejection [42, 43].

Recent approaches focused on the detection of CD3 expressing urinary EVs as carriers of messages originating directly from infiltrating T lymphocytes [44]. Specific mRNA multigene signatures were identified with a good specificity for acute rejection in a series of 192 urine samples collected simultaneously to indication biopsy. The signature revealed mRNA encoding for *CXCL11*, *CD74*, *IL32*, *STAT1*,*CXCL14*, *SERPINA1*, *B2M*, *C3*, *PYCARD*, *BMP7*, *TBP*, *NAMPT*, *IFNGR1*, *IRAK2*, and *IL18BP* [45]. In the same study, the authors identified another multigene signature (*CD74*, *C3*, *CXCL11*, *CD44*, and *IFNAR2*) that could distinguish TCMR from ABMR. Since RNA molecules are well protected inside the EVs, they can be assessed from urine samples even after prolonged storage, whereas proteins that are associated with the outer membrane of the EVs are exposed to protease activity and are less stable. mRNA evaluation, therefore, seems to offer the methodological advantages of an applicable biomarker.

The major limitations for a widespread diffusion of these biomarkers at the present time are mainly a lack of standardized procedures in isolation, purification, and characterization, which could potentially be solved by technological investment on commercially available kits. Another issue is the different experimental protocols adopted for assaying nucleic acids or proteins with different methodologies, rendering comparison of single studies and interpretation of results difficult. Moreover, no studies so far have included children (Fig. 1 and Table 1).

#### Urine transcriptomics

Urine mRNA transcripts were analyzed in several studies using different panels and approaches: in the CTOT-04 study, a set of urinary mRNAs (CD3ε, perforin, granzyme B, proteinase inhibitor 9, *CD103*, *IP-10*, *CXCR3*, transforming growth factor β1 [TGF-β1]) and 18S ribosomal RNA) was prospectively analyzed in 485 patients in the first year post-transplantation. A three-gene signature (*CD3ε*, interferon-inducible protein 10 (*IP-10*) formerly known as *CXCL10*, *18S* rRNA) was identified as able to discriminate ACR [46]. Moreover, analysis of the trajectories before the biopsy showing acute rejection demonstrated a marked increase in the three-gene signature starting from 120 days before rejection was clinically apparent. A significant modification in gene expression following adjustments to immunosuppressive protocol was also observed, suggesting that this signature may be useful in monitoring immune status [46]. In a further pilot study, still requiring validation, a 5-gene signature (*CD3ε*, *CD105*, *CD14*, *CD46*, and *18S* rRNA) further distinguished ACR from AMR [47].

Starting from biopsy material originating from different solid organ transplants, a common rejection module was identified and evaluated also in urine, where overexpression of *BASP1*, *CD6*, *CXCL10*, *CXCL9*, *INPP5D*, *ISG20*, *LCK*, *NKG7*, *PSMB9*, *RUNX3*, and *TAP1* was evidenced in the presence of acute rejection [48]. In other studies, such as the *CTOT-1*, where the previous mentioned panels were studied, the *CXCL9* chemokine and the corresponding protein alone were able to provide the best predictive value for diagnosing or excluding acute rejection. In that study, mRNA levels were highly sensitive and were an early marker of rejection, with levels rising at least 30 days before clinical signs [49]. In this context, the CRISPR–Cas13 method combined with the specific high-sensitivity enzymatic reporter unlocking (SHERLOCK) technology for the detection of *CXCL9* mRNA in urine is among the most attractive systems [50]. In this study, higher *CXCL9* mRNA levels are observed in samples from patients with biopsy-proven rejection compared with transplant recipients with no rejection or stable graft function, with a sensitivity of 93%. Furthermore, the assay, which could detect *CLCX9* at the attomolar range, was combined with the lateral flow-based system on a dipstick readable with a smartphone to allow quantification of band intensity. This advanced technique that allows accurate monitoring of *CXCL9* mRNA levels represents the most auspicious near future for a valuable biomarker, for its potential easy applicability, outside of transplant centers, where most of the patients are effectively being followed over time. However, the high potential of urinary mRNA assays is hampered by methodological limits due to mRNA degradation in urine, poor sample processing, and storage conditions for centralized analysis. Therefore, technology improvement is urgently needed to allow better standardization.

Other limited studies addressed microRNAs (miRNAs) as urinary biomarkers of rejection. MiRNAs are endogenous, single-stranded molecules of non-coding nucleotides able to repress the expression of target genes through the post-transcriptional degradation of mRNA and inhibition of protein expression. MiRNAs therefore retain an immunomodulant activity in innate and adaptive immune response. Lorenzen et al. studied a panel of 3 deregulated miRNAs and noted that levels of miR-10b and -210 were decreased and miR-10a increased in patients with acute renal allograft rejection. MiR-210 was predictive of GFR decline 1 year after transplantation and was able to differentiate patients with ACR from urinary tract infection [51].

Other miRNAs, including miR-142-3p, miR-210-3p, and miR-155-5p, were reported by Millàn et al. [52] to be deregulated in acute rejection. Among them, miR-155-5p proved to have the highest sensitivity and specificity and good negative and positive predictive values.

More recently, a combined approach of miRNAs [53] and urinary assay of *CXCL-9* and *CXCL-10* chemokine levels using a multivariable model evidenced that miR-155-5p, miR-615-3p, and *CXCL-9* levels were independent predictors of rejection. Levels of miR-155-5p, miR-126-3p, miR-21-5p, miR-25-3p, and miR-615-3p were significantly different between rejection and no-rejection urine; *CXCL-9* and *CXCL-10* protein levels were significantly elevated in urine from recipients with rejection, and the combination of these biomarkers produced a model with better diagnostic performance than the single biomarkers [53] (Fig. 1 and Table 1).

#### Urinary chemokines

Chemokines are secreted by leukocytes upon interferon γ stimulation and are critical regulators of leukocyte recruitment during allograft rejection. *CXCL10* and *CXCL9* upregulation was demonstrated by mRNA expression and protein synthesis increase in urine and blood in different models. Several studies of adults and 4 studies of children proved that urinary chemokines are valuable biomarkers for early detection of subclinical and clinical TCMR and for accurate monitoring of the response to treatment [54–57].

A recent multicenter, prospective study within the Predicting Renal transplant Outcome using BiomarkErs (PROBE) cohort demonstrated that *CXCL10*/Cr, assayed in urine paired with protocol biopsy, was significantly increased in rejection and declined upon treatment [58]. More interestingly, *CXCL10*/Cr rose 3–4 weeks prior to biopsy in cases with rejection, representing a potential tool for serial urinary surveillance and decision-making on biopsy timing and indication. The main limitation of the assay is its inability to distinguish between rejection and BKV infection or urinary infection.

The VIRTUUS prospective pediatric multicenter study is ongoing and aims to validate a urinary mRNA and metabolomics profile as diagnostic and prognostic biomarker of ACR using a urinary cell mRNA 3-gene signature including *CXCL10* mRNA [59] (Fig. 1 and Table 1).

#### Urinary metabolomics

Perturbations of kidney tissue metabolism can be assessed in urine with an unbiased “omic” approach known as “metabolomics”. This mass spectrometry-based method simultaneously measures multiple metabolites. The aim, adopted by independent groups, was to define a distinct and specific metabolic signature for rejection and other kidney diseases.

In a recent study performed in a pediatric kidney transplant cohort [60] including 30 TCMR and 54 borderline tubulitis, ten metabolites, namely proline, kynurenine, sarcosine, methionine sulfoxide, threonine, glutamine, phenylalanine, alanine, and PC.aa.C34.4 and PC.ae.C38.6, produced by activated macrophages and T helper 1 subsets, displayed a good correlation with cellular rejection. The study however has some methodological limitations and needs to be confirmed in other patient cohorts.

The pilot study of Banas et al. [61] defined in NMR-spectroscopy another set of candidate markers, namely, alanine, citrate, dimethylamine (DMA), glucose, glucuronate, hippurate, lactate, phenylacetylglutamine (PAQ), trigonelline, and urea, able to discriminate rejection. Metabolites such as lactate may represent the downstream products of cellular activity and mitochondrial derangement as in tubular damage, and it is of note that no soluble metabolites of clear lymphocyte origin were identified.

The same methodological approach is now being applied in a multicenter cohort comprising 972 histologically and clinically characterized patients in the PARASOL study [62], aiming at validating a metabolic signature of rejection with good predictive value.

Very promising is the employment of combined “omics” such as the QSant assay [63], which integrates the measurement of urinary cell-free DNA, methylated-cell-free DNA, total proteins, *CXCL-10*, clusterin, and creatinine. By using an artificial intelligence algorithm, results are expressed as a Q score ranging from 0 to 100, which discriminates with a good predictive value between acute rejection and no rejection (Fig. 1 and Table 1).

#### Urinary proteomics

Urine proteomics, similar to the other “omic” sciences, relies on a large-scale study approach without the bias of a hypothetical selection of urinary proteins. Concerning a complex phenomenon like rejection, the amount of the generated data and the different experimental conditions have produced interesting results to date, but not a generalized consensus toward the choice of the different profiles.

One of the most interesting contributions in children identified by capillary electrophoresis-mass spectrometry (CE-MS) is a urinary proteomic profile of 79 proteins able to distinguish with good performance chronic active ABMR and was validated also in an independent cohort [64, 65]. It is of note that most of the proteins were fragments of collagen, alpha-1-antitrypsin, retinol-binding protein 4, fibrinogen alpha chain, neurosecretory protein VGF, Ig kappa chain C region, beta-2-microglobulin, and annexin A, but that no immunologically active proteins were detected.

Another recent study [66] described a panel of the ten most-represented proteins (alpha-1 B glycoprotein, afamin, apolipoprotein A1, apolipoprotein A4, Ig heavy constant a1, Ig heavy constant g4, leucine rich a2 glycoprotein 1, alpha-1 antitrypsin, antithrombin and transferrin) able to discriminate ABMR with good sensitivity and specificity using nano-reversed–phase liquid chromatography and shotgun mass spectrometry. These proteins were already described in other settings of kidney disease and are mainly reflecting general injury or injury mechanisms, rather than a specifically immunologically mediated process. This assay displayed a good negative predictive performance and was proposed as a screening tool for early diagnosis of ABMR with good confidence.

Urinary proteomic biomarkers are indeed very promising. However, until now, they have yet to reach widespread diagnostic use, which will likely rely on technology improvements and assay standardization (Fig. 1 and Table 1).

### Non-invasive blood biomarkers

A large number of blood biomarkers have been studied using different methodologies, but only a few have reached clinical practice with assays available beyond the investigational context.

#### Donor-derived cell-free DNA

The term cell-free DNA (cfDNA) defines DNA fragments approximately 100–200 bp long, which are generated and released into the circulation by cells undergoing apoptosis. While this is a physiologic process, the amount of cfDNA may increase in conditions of cellular damage. In the field of transplantation, cellular injury of the graft derived from immune cell attack should result in a net increase of the amount of cfDNA of donor origin. In line with the notion that cfDNA is a marker of cellular injury, a rise in cfDNA may also be reflective of other causes of allograft injury, i.e., infection and acute tubular injury. Quantification of cfDNA of donor origin may therefore represent an effective and minimally invasive way to monitor rejection. In general, a positive correlation between high levels of donor-derived cfDNA (dd-cfDNA) and development of acute rejection was reported for lung, liver, heart, and kidney, including in pediatric recipients [67].

Even though this marker is promising, several areas of uncertainty remain. The first one concerns how to technically detect cfDNA: the principle behind dd-cfDNA quantification relies on the genetic differences between donor and recipient. The most accurate and sensitive methods are based on the analysis of panels of dozens or hundreds of single nucleotide polymorphisms (SNPs) performed using next-generation sequencing (NGS) approaches, which are quantitative. However, these methods are presently characterized by a long turnaround time of analysis and high costs, therefore limiting its application in the routine management of transplanted patients.

Given its high specificity and sensitivity in detecting donor DNA in a recipient’s blood, droplet digital PCR (ddPCR) is a valid alternative to NGS, providing a quantitative approach with contained costs [68].

The second area of uncertainty concerns how to quantify cfDNA and how to define an actionable threshold. The most common way to quantify cfDNA of the donor is as a percentage of the total. While this is a convenient method, it may be troublesome in the presence of infections, which can cause damage to the recipient affected cells and tissues, thereby increasing the total amount of recipient cfDNA and causing an underestimation of cfDNA of donor origin. The issue of the threshold remains perhaps the most critical point in the transfer of the assay in a clinical setting. In general, thresholds are dependent on the “total transplanted mass.” Consistently, the percentage of dd-cfDNA in liver transplanted patients is the highest, followed by lung, kidney, and heart.

The third area of uncertainty concerns how to use cfDNA: the issue of the value of cfDNA to monitor transplant rejection remains debatable [67]. In general, the great majority of studies highlight a positive correlation between the amount of cfDNA of donor origin and the presence of acute rejection. In addition, there seems to be an increase in dd-cfDNA levels in patients developing de novo DSA, suggesting that combined monitoring of dd-cfDNA and dnDSA may be more accurate in identifying patients undergoing ABMR and may help refine the patient population benefitting from a bioptic study of the graft [69].

Lastly, it has to be noted that the use of cell-free DNA in children poses additional problems, linked to the possibility of obtaining enough material from liquid biopsies and to optimizing cut-offs for this population. Recent data, however, have confirmed that in heart transplant recipients, children behave similarly to adults, with cfDNA being a very accurate predictor of rejection episodes [70, 71]. The experience with kidney transplants is far more limited, and more studies with larger patient cohorts and longer follow-up are needed determine the impact of dd-cfDNA monitoring during routine follow-up of kidney-transplanted patients (Fig. 1 and Table 1).

#### Peripheral blood transcriptomics

Peripheral blood gene expression profiling has also been used in independent studies to highlight a minimum set of genes that can predict rejection. In the Kidney Allograft Immune Biomarkers of Rejection Episodes (KALIBRE) study, Christakoudi and colleagues [72] studied by RT-qPCR the expression of 22 literature-based genes in peripheral blood samples from 248 patients. A 7-gene TCMR signature (*IFNG*, *IP-10*, *ITGA4*, *MARCH8*, *RORc*, *SEMA7A*, *WDR40A*) predicted rejection 7 weeks in advance of traditional markers. Furthermore, resolution of the rejection episode in response to therapy resulted in the return of gene expression levels to baseline values. Another study identified a 17-gene peripheral blood signature in patients with subclinical TCMR who received a protocol biopsy 3 months post-transplant within the GoCAR study (Genomics of Chronic Allograft Rejection) [73]. The targeted expression assay (TREx), validated in an external cohort, proved to have a high positive and negative predictive value over graft loss at 24 months.

In the multicenter prospective BIOMARGIN study, an 8-gene assay (*CXCL10*, *FCGR1A*, *FCGR1B*, *GBP1*, *GBP4*, *IL15*, *KLRC1*, *TIMP1*) was defined in a cohort of 49 patients with ABMR. This panel, validated in an independent cohort, proved to be of good diagnostic accuracy for times of stable graft function and of graft dysfunction, in the first and subsequent years. The 8-gene assay showed a good correlation with microvascular inflammation and transplant glomerulopathy, but not with the histological grade of the TCMR lesions [74].

The k-SORT (Kidney Solid Organ Response Test) 17-gene signature was defined in the multicenter Assessment of Acute Rejection in Renal Transplant (AART) study, also including a cohort of children: this assay proved predictive with a 93% positive predictive value, of both TCMR and ABMR 3 months before biopsy in the discovery and the validation cohort [75]. In another recent study, retrospectively recruiting 1763 samples from a multicenter biobank, the k-SORT assay did not reach the diagnostic value displayed in the discovery cohort [76].

The CTOT-08 prospective multicenter trial enrolled adults on a surveillance biopsy protocol and used a 57-gene expression profile signature that efficiently discriminated between stable grafts without histological signs of rejection and subclinical rejection at 24 months with good accuracy. Thanks to the elevated negative predictive value for subclinical acute rejection (NPV 88%), this assay would potentially allow avoiding unnecessary biopsies in patients with negative results [77]. These data were confirmed in an independent study [78]. This test is available as TruGraf® and is performed by the producer (Eurofins-Viracor, Transplant Genomics Inc. USA). However, to date, it has not been cleared or approved for diagnostic use by the US Food and Drug Administration (Fig. 1 and Table 1).

#### Blood proteomics

The identification of blood proteins with biomarker significance requires a high resolving fractionating method, to be able to fish informative proteins at extremely low concentrations and overcome the confounding prevailing presence of plasma proteins deriving from normal tissue homeostasis.

Cibrik et al. [79] identified a “protein signature” able to discriminate between stable transplant patients and those with rejection. Of the 17 proteins that define the signature, in the rejecting cohort, nine had lower expression (E-cadherin, EGF, erythropoietin receptor, growth regulated oncogene-alpha, interleukin 6, MCP-1 macrophage inflammatory protein 3-alpha, transforming growth factor β 1 and 2) and eight higher (GM-CSF, interleukin 1 receptor 1, interleukin 12 p70, KIM-1, MIF, osteopontin, tumor necrosis factor receptor II, and vascular endothelial growth factor receptor). While interesting, this approach remains inconclusive due to the methodological limitations of a cohort study on indication biopsy and the choice of protein set based on the available antibodies. Other studies, including retrospective cohorts studied with a combination of IL-1 receptor antagonist, IL-20, and sCD40L or panels of other proteins or soluble co-stimulatory molecules such as CD30, are extensively reviewed elsewhere (Fig. 1 and Table 1).

#### Peripheral blood EVs

Extracellular vesicles (EVs) derived from plasma have been investigated so far only in a few non-cross-validated studies [40] due to the higher technical complexity required for proper characterization, being > 70% of lymphoid origin and possibly masking signals derived from graft cells.

In a small cohort study of 28 adults, plasma density of EVs with surface expression of C4d/CD144, as marker of endothelial injury, was associated with ABMR, correlated to biopsy severity and was modulated by rejection treatment [80]. Another small study of 64 adult patients (18 ABMR and 8 TCMR) identified in plasma EVs a gene expression combination score of 4 genes (*gp130*, *SH2D1B*, *TNFα*, and *CCL4*) significantly higher in the ABMR than TCMR subjects [81]. While EVs appear a versatile tool to explore the immunological and inflammatory mechanisms of allorecognition, methodological complexities need to be addressed before they can be considered a widely applicable biomarker (Fig. 1 and Table 1).

#### Markers of immune status: virus specific T cells and torque teno virus

The assessment of virus-specific T cells (Tvis) is a functional marker of immunosuppression that was prospectively investigated in a pediatric phase 2 randomized multicenter study. Serial assessment of CD4 Tvis against adenovirus, cytomegalovirus, and herpes symplex virus allowed personalized steering of immunosuppressive therapy with a net reduction of the exposure to unnecessary overimmunosuppression, reduced infection events, and comparable kidney function [82].

Another approach, leading to conceptually similar information, is the quantitative assay of torque teno virus (TTV) load, which allows one to simplify the complex laboratory technique used for Tvis analysis. TTV is a non-pathogenic virus present in the majority of individuals, with increasing replication in parallel to the increase of immunosuppression status. TTV load can represent a direct biomarker of the immune status, being potentially useful to steer drug dosage to target the proper balance between rejection and excess infection, with good performance [83]. Two randomized prospective interventional trials are ongoing.

## Conclusions

Allorecognition is a complex phenomenon that cannot be thoroughly captured by the traditional biomarkers in use, which measure organ function, but generally fail to predict rejection before it becomes clinically evident. Recent experimental approaches have highlighted novel possibilities that will allow us to monitor graft health, modulate levels of immunosuppression, and ultimately to eliminate or reduce the need for protocol biopsies.

Even if these approaches have been successful in several clinical trials, they still need to reach wide clinical application. This is particularly true in the pediatric setting, where the small patient numbers often preclude systematic testing of novel assays to diagnose rejection. Large and validated studies, addressing also the pediatric population, are therefore needed to take advantage of these highly versatile tools non-invasively and longitudinally over the long run.

## Supplementary information

Below is the link to the electronic supplementary material.

Supplementary file1 (DOCX 15 KB)

Supplementary file2 (DOCX 46 KB)
