# Supplementary material for: Rejection markers in kidney transplantation: do new technologies help children?
Source: Pediatr Nephrol. 2023 Jan 17;38(9):2939–55. doi: 10.1007/s00467-022-05872-z (PMC10432336; doi:10.1007/s00467-022-05872-z)
Supplement: Supplementary file 1 — Supplementary file1 (DOCX 15 KB) [file 467_2022_5872_MOESM1_ESM.docx]

**Supplementary Table 1**

Biomarker definition and characteristics according to BEST (Biomarkers, EndpointS, and other Tools) Resource and potential meaning in transplantation [1]

| Type of biomarker (BEST) | Definition  (BEST) | Explanation  (BEST) | Performance/ examples | Potential meaning in transplantation |
| --- | --- | --- | --- | --- |
| **Diagnostic biomarker** | A biomarker used to detect or confirm presence of a disease or condition of interest or to identify individuals with a subtype of the disease | used for the critical determination of whether a patient has a particular medical condition  accurate diagnosis warrants assessment of the clinical performance of diagnostic biomarker | **Sensitivity:** ability of a screening test to detect a true positive  **Specificity:** ability of a screening test to detect a true negative  **Positive predictive value (PPV)** :  proportion of those who tested positive who actually have the disease or condition  **Negative predictive value (NPV)**:  proportion of those who tested negative who actually do not have the disease or condition. | Identifies patient with a condition: i.e.  graft biopsy identifies acute rejection |
| **Monitoring biomarker** | A biomarker measured repeatedly for assessing status of a disease or medical condition or for evidence of exposure to (or effect of) a medical product or an environmental agent | Used to monitor disease progression, worsening of previously existing abnormalities, or change in disease severity or specific abnormalities  Used to monitor response of a disease or condition to a treatment | Changes in biomarker measurements observed during or after treatment may provide supporting evidence of a pharmacodynamic effect or an early therapeutic response  Safety biomarker measured repeatedly in early phase clinical trials can be a type of monitoring biomarker for organ toxicity  **Sensitivity**: ability of a screening test to detect a true positive  **Specificity:** ability of a screening test to detect a true negative | Serially measured to detect changes in the graft:  Proteinuria monitoring  Tacrolimus levels to detect exposure to immunosuppressive drugs  Signs of drug toxicity |
| **Response biomarker** | A biomarker used to show that a biological response, potentially beneficial or harmful, has occurred in an individual who has been exposed to a medical product or an environmental agent | Pharmacodynamic biomarker: A response biomarker that indicates biologic activity of a medical product  Surrogate endpoint biomarker: A response biomarker that is an endpoint used in clinical trials as a substitute for a direct measure of how a patient feels, functions, or survives. | used to show that a biological response, potentially beneficial or harmful, has occurred in an individual who has been exposed to a medical product  **Sensitivity**: ability of a screening test to detect a true positive  **Specificity:** ability of a screening test to detect a true negative | Verifies that a biological response has occurred after a treatment:  Serum Creatinine reduction upon rejection treatment  Lympohcyte reduction upon thymoglobulin use  DSA MFI reduction after treatment of ABMR |
| **Predictive biomarker** | A biomarker used to identify individuals more likely than similar individuals without the biomarker to experience a favorable or unfavorable effect from exposure to a medical product | used to identify individuals who are more likely to respond to exposure to a particular medical product | **Sensitivity:** ability of a screening test to detect a true positive  **Specificity:** ability of a screening test to detect a true negative  **Positive predictive value (PPV)** :  proportion of those who tested positive who actually have the disease or condition  **Negative predictive value (NPV)**:  proportion of those who tested negative who actually do not have the disease or condition | estimates the likelihood of achieving an outcome:  IF/TA predictive of renal function decline |
| **Prognostic biomarker** | A biomarker used to identify likelihood of a clinical event, disease recurrence or progression in patients who have the disease or medical condition of interest. | A prognostic biomarker indicates an increased (or decreased) likelihood of a future clinical event, disease recurrence or progression in an identified population. | **Sensitivity:** ability of a screening test to detect a true positive  **Specificity:** ability of a screening test to detect a true negative  **Positive predictive value (PPV)** :  proportion of those who tested positive who actually have the disease or condition  **Negative predictive value (NPV)**:  proportion of those who tested negative who actually do not have the disease or condition | estimates the likelihood of a clinical event or of disease progression, staging severity of disease  severe rejection with risk of graft loss |
| **Safety biomarker** | A biomarker measured before or after an exposure to a medical product to indicate the likelihood, presence, or extent of toxicity as an adverse effect. | ability to detect or predict these adverse drug. In some cases, the toxicity is signaled by the detection of or change in a biomarker, allowing dose modification or treatment interruption before toxicity becomes severe | **Sensitivity**: ability of a screening test to detect a true positive  **Specificity:** ability of a screening test to detect a true negative | Drug level in target  Lymphocyte count upon T cell depleting induction |
| **Susceptibility/Risk biomarker** | A biomarker that indicates the potential for developing a disease or medical condition in an individual who does not currently have clinically apparent disease or the medical condition. |  | **Sensitivity:** ability of a screening test to detect a true positive  **Specificity:** ability of a screening test to detect a true negative  **Positive predictive value (PPV)** :  proportion of those who tested positive who actually have the disease or condition  **Negative predictive value (NPV)**:  proportion of those who tested negative who actually do not have the disease or condition | EBV negative serology: risk of developing primary EBV infection and EBV post transplant proliferative disease |

Additional Reference

[1] FDA-NIH Biomarker Working Group. BEST (Biomarkers, EndpointS, and other Tools) Resource [Internet]. Silver Spring (MD): Food and Drug Administration (US); 2016-. Co-published by National Institutes of Health (US), Bethesda (MD)
