# Supplementary material for: Rejection markers in kidney transplantation: do new technologies help children?
Source: Pediatr Nephrol. 2023 Jan 17;38(9):2939–55. doi: 10.1007/s00467-022-05872-z (PMC10432336; doi:10.1007/s00467-022-05872-z)
Supplement: Supplementary file 2 — Supplementary file2 (DOCX 46 KB) [file 467_2022_5872_MOESM2_ESM.docx]

**Supplementary Table 2**

Studies assessing Tacrolimus IPV in children and adults

| **Reference** | **Study population** | **IPV Assessment period** | **Unit** | **Tac IPV cut-off** | **Outcome**  **DSA development** | **Outcome Rejection** | **Outcome**  **Graft loss** |
| --- | --- | --- | --- | --- | --- | --- | --- |
| Prytula et al. Pediatr Transpl (2012) 16:613–618. <https://doi.org/10.1111/j.1399-3046.2012.01727.x> [1] | 113 children | 6-12 months | CV SD/mean x100 | quartile | Not assessed | CV 48.7% in rejecting vs 32.9% in no rejection p=0.045  Not significant difference at 24-36 months | CV 34.2% in graft loss vs 31.9% in functioning NOT SIGNIFICANT |
| Pizzo et al. Pediatr Nephrol (2016) 31:2345–2352. <https://doi.org/10.1007/s00467-016-3422-5> [2] | 23 children | 6 months | CV SD/mean x100 | 31% | Significant association to DSA | Significant association to rejection | Not assessed |
| Solomon et al. Pediatric Nephrology (2020) 35:261–270. <https://doi.org/10.1007/s00467-019-04377-6> [3] | 38 children | > 3 months | CV SD/mean x100 | 30%  40%  50% | 19(50%) de novo DSA at median 1.2 years (0.6-29y). Although not statistically significant, higher tacrolimus variability was associated with increased de novo DSA development at all three CV cut-points(≥30%:20% vs 55%; ≥40%:36% vs 58%; ≥50%:46% vs 57%; tables 1,2 and 3) | IPV>30% 42% rejection  Not significant | Not significant |
| Abu Bakar et al. Pediatr Nephrol (2019) 34:2557–2562. <https://doi.org/10.1007/s00467-019-04346-z> [4] | 25 children | 12 months | CV SD/mean x100 +  mean absolute deviation | 26% | Tacrolimus IPV was calculated using two methods, CV and MAD. Rejecters had higher CV (median, IQR 0.44, 0.36-0.61 v. 0.24, 0.19-0.35, p<0.0001) and higher MAD (0.33, 0.25-0.48 v. 0.19, 0.15-0.26) | Rejecters High CV> 0.44 (0.36-0.61 vs 0.24 (0.19-0.35 p<0.0001 |  |
| Kaya Aksoy et al. Eur J Drug Metab Pharmacokinet (2019) 44:539–548. <https://doi.org/10.1007/s13318-019-00544-0> [5] | 67 children | 6-12 months | CV SD/mean x100 | 32% | At 6 months CV>32%: 67% DSA vs 31% no DSA p=0.027  At 12 months CV>50% 83% vs 47% p=0.0033  HR CV>32% 0.204 (0.0370.897) p =0.044 |  |  |
| Gold et al. Transpl Int (2020) 33(12):1681-1692.  <https://doi.org/10.1111/tri.13726> [6] | Multicenter  1419 children | 1-2 years | Maximum ratio between 2 consecutive levels IPV 30% = 1.54  IPV 45% ratio 1.93 | Ratio 1.5- 2.0 | Not reported | Non reported | Ratio >1.5  Increased risk of graft loss at 3-5 years  P<0.05 for all ages  0-11 years  HR 3.86 (1.03-14.4) p 0.045  12-23 years  HR 2.47 (1.57-3.87) p<0.001  23-34 years  HR 1.76 (1.14-2.74) p<=.012 |
| Choi et al. Pediatr Transplant (2022) 26(6):e14297.  <https://10.1111/petr.14297> [7] | 202 children | 6-12 months | CV SD/mean x100 | 27.67% | High IPV (>27.67%) HR 2.408 (1.34.4.312) p=0.003 | High IPV (>27.67%) HR 5.571 (2.176-14..262) p<0.001 | High IPV (>27.67%) HR 10.519 (1.179-94.363) p=0.036 |
| Baghai Arassi et al. Pediatr Nephrol (2022) 37:2503–2514 <https://doi.org/10.1007/s00467-022-05426-32022> [8] | 48 children |  | CV 6-12 months post tx | 25% | 31% DSA at median 37 months (IQR 3-50)  HR 3.4 (1.0-11-1) p0.047 1 year post tx | HR 4.1 (95CI 1.1-14-8) p=0.033 | Not significant |
| Borra et al. Nephrol Dial Transpl (2010) 25:2757–2763 <https://doi.org/10.1093/ndt/gfq096> [9] | 297 Adults | 6-12 months |  | Mean 17%  Median 14.9% | Not assessed | Composite end point p<0.001 | Composite end point p<0.001 |
| Shuker et al. Transpl Int (2016) 29(11):1158-1167  <https://doi.org/10.1111/tri.12798> [10] | 808 Adults | 6-12 months |  | Median 16.2% | Not assessed | Composite end point p 0.018 |  |
| Rodrigo et al. Transplantation (2016) 100:2479–2485. <https://doi.org/10.1097/TP.0000000000001040> [11] | 310 Adults | 4-12 months |  | Mean 30% | Not assessed | HR 2.5 (1.26-5.5) p=0.009 | Cv>30% HR 2.92 (1.47-5.8) p=0.045 |
| Whalen et al. Transplantation (2017) 101(2):430-436  <https://doi.org/10.1097/TP.0000000000001129> [12] | 376 Adults | 6-12 months |  | Mean 15% | Not assessed | HR 1.95 (1.23-3.09) p=0054 | HR 4.34 (1.25-15.10) p=0.0207 |
| Goodall et al. Transplant Direct (2017) 7;3(8):e192.  <https://doi.org/10.1097/TXD.0000000000000710> [13] | 328 Adults | 6-12 months |  | Quartiles lowest 13.5% Highest 25.27% | Not assessed | HR 9.83 (4.62-30.94) P<0.0001 | 2.51 (1.01-6.27) p= 0.048 |
| Taber et al Transplantation (2017) 101(12):2931-2938.  <https://doi.org/10.1097/TP.0000000000001840> [14] | 1411 Adults | 1 month until event |  | 40% | Not assessed | HR 1.2 (1.13-1.28) | HR 1.30 (1.23-1.37) |
| Siebert et al. Clin Transplant (2018) 32(12):e13424.  <https://doi.org/10.1111/ctr.13424> [15] | 1226 Adults | 0-6 months |  | quartiles | Not assessed |  | Highest quartile (49%) HR 2.95 (1.67-5.23) p=0.0002 |
| Susal et al. Am J Transplant (2019) 19(10):2805-2813  <https://doi.org/10.1111/ajt.15346> [16] | 6638 Adults | 1-3 years |  | Tertiles 30% 44%  >45% | Not assessed |  | IPV 30-44% HR 1.42 (1.11-1.82) p=0-0005  IPV >45% HR 2.11 (1.61-2.77) p<0.0001 |

**REFERENCES**

1. Prytula AA, Bouts AH, Mathot RA, van Gelder T, Croes LK, Hop W, Cransberg K (2012) Intra-patient variability in tacrolimus trough concentrations and renal function decline in pediatric renal transplant recipients. Pediatr Transplant 16:613-618.

2. Pizzo HP, Ettenger RB, Gjertson DW, Reed EF, Zhang J, Gritsch HA, Tsai EW (2016) Sirolimus and tacrolimus coefficient of variation is associated with rejection, donor-specific antibodies, and nonadherence. Pediatr Nephrol 31:2345-2352.

3. Solomon S, Colovai A, Del Rio M, Hayde N (2020) Tacrolimus variability is associated with de novo donor-specific antibody development in pediatric renal transplant recipients. Pediatr Nephrol 35:261-270.

4. Abu Bakar K, Mohamad NA, Hodi Z, McCulloch T, Williams A, Christian M, Key T, Kim JJ (2019) Defining a threshold for tacrolimus intra-patient variability associated with late acute cellular rejection in paediatric kidney transplant recipients. Pediatr Nephrol 34:2557-2562.

5. Kaya Aksoy G, Comak E, Koyun M, Akbas H, Akkaya B, Aydinli B, Ucar F, Akman S (2019) Tacrolimus Variability: A Cause of Donor-Specific Anti-HLA Antibody Formation in Children. Eur J Drug Metab Pharmacokinet 44:539-548.

6. Gold A, Tonshoff B, Dohler B, Susal C (2020) Association of graft survival with tacrolimus exposure and late intra-patient tacrolimus variability in pediatric and young adult renal transplant recipients-an international CTS registry analysis. Transpl Int 33:1681-1692.

7. Choi JS, Ko H, Kim HK, Chung C, Han A, Min SK, Ha J, Kang HG, Ha IS, Min S (2022) Effects of tacrolimus intrapatient variability and CYP3A5 polymorphism on the outcomes of pediatric kidney transplantation. Pediatr Transplant 26:e14297.

8. Baghai Arassi M, Gauche L, Schmidt J, Hocker B, Rieger S, Susal C, Tonshoff B, Fichtner A (2022) Association of intraindividual tacrolimus variability with de novo donor-specific HLA antibody development and allograft rejection in pediatric kidney transplant recipients with low immunological risk. Pediatr Nephrol 37:2503-2514.

9. Borra LC, Roodnat JI, Kal JA, Mathot RA, Weimar W, van Gelder T (2010) High within-patient variability in the clearance of tacrolimus is a risk factor for poor long-term outcome after kidney transplantation. Nephrol Dial Transplant 25:2757-2763.

10. Shuker N, Shuker L, van Rosmalen J, Roodnat JI, Borra LC, Weimar W, Hesselink DA, van Gelder T (2016) A high intrapatient variability in tacrolimus exposure is associated with poor long-term outcome of kidney transplantation. Transpl Int 29:1158-1167.

11. Rodrigo E, Segundo DS, Fernandez-Fresnedo G, Lopez-Hoyos M, Benito A, Ruiz JC, de Cos MA, Arias M (2016) Within-Patient Variability in Tacrolimus Blood Levels Predicts Kidney Graft Loss and Donor-Specific Antibody Development. Transplantation 100:2479-2485.

12. Whalen HR, Glen JA, Harkins V, Stevens KK, Jardine AG, Geddes CC, Clancy MJ (2017) High Intrapatient Tacrolimus Variability Is Associated With Worse Outcomes in Renal Transplantation Using a Low-Dose Tacrolimus Immunosuppressive Regime. Transplantation 101:430-436.

13. Goodall DL, Willicombe M, McLean AG, Taube D (2017) High Intrapatient Variability of Tacrolimus Levels and Outpatient Clinic Nonattendance Are Associated With Inferior Outcomes in Renal Transplant Patients. Transplant Direct 3:e192.

14. Taber DJ, Su Z, Fleming JN, McGillicuddy JW, Posadas-Salas MA, Treiber FA, Dubay D, Srinivas TR, Mauldin PD, Moran WP, Baliga PK (2017) Tacrolimus Trough Concentration Variability and Disparities in African American Kidney Transplantation. Transplantation 101:2931-2938.

15. Seibert SR, Schladt DP, Wu B, Guan W, Dorr C, Remmel RP, Matas AJ, Mannon RB, Israni AK, Oetting WS, Jacobson PA (2018) Tacrolimus trough and dose intra-patient variability and CYP3A5 genotype: Effects on acute rejection and graft failure in European American and African American kidney transplant recipients. Clin Transplant 32:e13424.

16. Susal C, Dohler B (2019) Late intra-patient tacrolimus trough level variability as a major problem in kidney transplantation: A Collaborative Transplant Study Report. Am J Transplant 19:2805-2813.
